# Isolation and Staining Reveal the Presence of Extracellular DNA in Marine Gel Particles

**DOI:** 10.3390/gels9030251

**Published:** 2023-03-21

**Authors:** Aisha S. M. Al-Wahaibi, Robert C. Upstill-Goddard, J. Grant Burgess

**Affiliations:** 1 Centre of Excellence in Marine Biotechnology, Sultan Qaboos University, P.O. Box 50, Muscat 123, Oman; 2 School of Natural and Environmental Sciences, Newcastle University, Newcastle upon Tyne NE1 7RU, UK

**Keywords:** marine gel particles, extracellular polymeric substances, transparent exopolymeric particles, Coomassie stained particles, extracellular DNA, staining, bioimaging, *Pseudoalteromonas atlantica*

## Abstract

Marine gel particles (MGP) are amorphous hydrogel exudates from bacteria and microalgae that are ubiquitous in the oceans, but their biochemical composition and function are poorly understood. While dynamic ecological interactions between marine microorganisms and MGPs may result in the secretion and mixing of bacterial extracellular polymeric substances (EPS) such as nucleic acids, compositional studies currently are limited to the identification of acidic polysaccharides and proteins in transparent exopolymer particles (TEP) and Coomassie stainable particles (CSP). Previous studies targeted MGPs isolated by filtration. We developed a new way of isolating MGPs from seawater in liquid suspension and applied it to identify extracellular DNA (eDNA) in North Sea surface seawater. Seawater was filtered onto polycarbonate (PC) filters with gentle vacuum filtration, and then the filtered particles were gently resuspended in a smaller volume of sterile seawater. The resulting MGPs ranged in size from 0.4 to 100 µm in diameter. eDNA was detected by fluorescent microscopy using YOYO-1 (for eDNA), with Nile red (targeting cell membranes) as a counterstain. TOTO-3 was also used to stain eDNA, with ConA to localise glycoproteins and SYTO-9 for the live/dead staining of cells. Confocal laser scanning microscopy (CLSM) revealed the presence of proteins and polysaccharides. We found eDNA to be universally associated with MGPs. To further elucidate the role of eDNA, we established a model experimental MGP system using bacterial EPS from *Pseudoalteromonas atlantica* that also contained eDNA. Our results clearly demonstrate the occurrence of eDNA in MGPs, and should aid furthering our understanding of the micro-scale dynamics and fate of MGPs that underly the large-scale processes of carbon cycling and sedimentation in the ocean.

## 1. Introduction

Marine gel particles (MGPs) are ubiquitous organic particles comprising biopolymers that can be released from marine bacteria, diatoms, macroalgae and some marine invertebrates. MGPs vary in composition and size from nanogels (100–150 nm) < microgels (~5 µm) < macrogels (100 µm) < marine snow (>500 µm to 10s of cm) [1]. Transparent exopolymer particles (TEP) and Coomassie stainable particles (CSP) are well known MGP components [2,3,4] that principally comprise extracellular polymeric substances (EPS) able to self-assemble naturally [5,6,7]. The adhesive nature of MGPs leads them to form suspended aggregates (flocs), implicating them in ocean carbon sequestration [8,9,10] via the formation of “marine snow” [11]. MGPs also contribute to problematic biofilm formation via surface attachment. Examples include biofilms on desalination plant reverse osmosis (RO) membranes and ship hulls [12,13,14,15]. Indeed, MGPs have been referred to as ‘protobiofilms’ [14], reflecting their similarities with biofilms in terms of structure, and their biochemical and physiological properties. While the biochemical composition of attached biofilms has been widely studied [16,17,18,19,20], corresponding studies on MGPs, despite their potential global relevance, are relatively rare.

Studies of the biochemical composition of MGPs mainly involve staining them with either Alcian blue or Coomassie brilliant blue dyes, with the subsequent identification of particles comprising polysaccharides in TEP and proteins in CSP [21,22,23,24,25]. These methods are straightforward, but they allow only limited insight into MGP origin, structure, and function.

Elucidating the precise origin of MGPs is challenging due the diverse origins of the gel-like material that include diatoms, bacteria, cyanobacteria and seagrass [26,27,28,29,30]. Their rapid adherence, aggregation and dispersal can further confound studies of their composition. Previously MGPs were examined after isolation on filters and/or in samples fixed to microscope slides, but this can hinder a more complete understanding of their natural properties in three-dimensional space [21,31,32,33]. One study used an in situ FlowCAM to enable real-time TEP quantification [34], but this has limited applicability to samples collected in remote locations, such as on a research cruise.

In attached marine biofilms, dynamic ecological interactions between marine microorganisms can result in the secretion of extracellular DNA (eDNA) that likely enhances bacterial attachment and biofilm structural integrity [16,18,35]. If eDNA is similarly secreted by bacteria residing in MGPs, it could potentially affect MGP aggregation and sedimentation. Studies of eDNA in biofilms commonly use fluorescent staining coupled with confocal laser scanning microscopy (CLSM) [36,37,38], a technique that is also well-established for characterising the composition of hydrated EPS matrices and marine snow [39,40,41,42,43]. To help identify eDNA within MGP, fluorescent intracellular DNA (iDNA) dyes have been used to visualise particle-associated marine bacteria. These dyes penetrate the cell membrane and bind to DNA, allowing differentiation between iDNA and eDNA [44,45,46,47].

In this study, our aim was to develop a new technique to enable the separation of MGPs from natural seawater as intact suspended particles, facilitating their characterisation in three-dimensional space. We subsequently interrogated MGPs for the presence of eDNA using fluorescent dyes and CLSM. Given that bacteria are important MGP producers [3,12], we also devised a simple model system to aid understanding the composition and behaviour of bacterially derived MGPs. This is based on *Pseudoalteromonas atlantica*, a widespread and well-characterised marine bacterium known to produce extracellular polymeric substances (EPS) and biofilms, and to contribute to MGP aggregation [48,49,50].

## 2. Results

### 2.1. Microscopy of Purified Natural MGPs in Liquid Suspension

**Figure 1:**
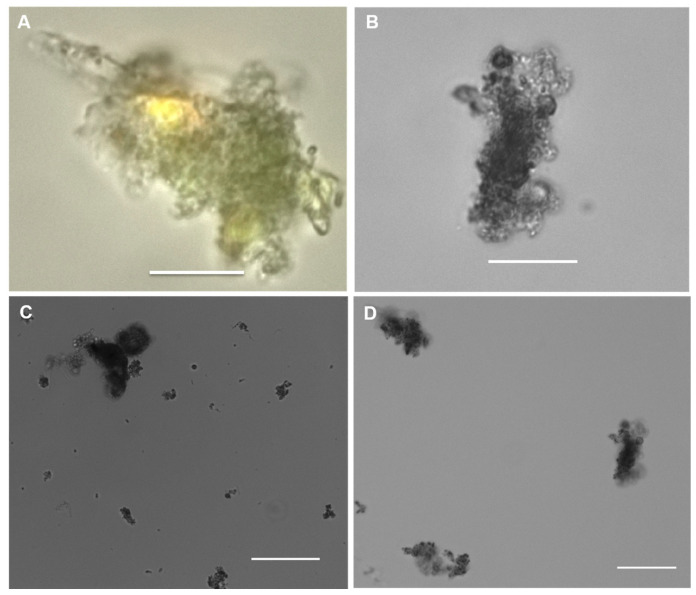
Marine gel particles recovered from PC filters after re-suspension in 0.2 µm filter sterilised seawater. Scale bar = 20 µm, image captured at 63× objective and visualised with phase contrast (**A**). Scale bar = 50 µm (**B**–**D**), image captured at 40× objective for (**B**) and 20× for (**C**,**D**) and visualised using bright-field microscopy.

MGPs ranging from 0.4 to 100 µm in diameter were obtained from the polycarbonate filter (PC filter) suspension. MGPs were visualised using various objective lenses (Figure 1A–D). The particles were concentrated 200-fold by resuspending them from one litre of seawater into a volume of 5 mL of sterile seawater. The particles were of various sizes and shapes, including porous, sheet-like, and filament-like. The particles ranged from grey to dark black as they contained different materials such as faecal pellets and detritus (Figure 1A).

### 2.2. Visualisation of the Natural MGP Matrix

**Figure 2:**
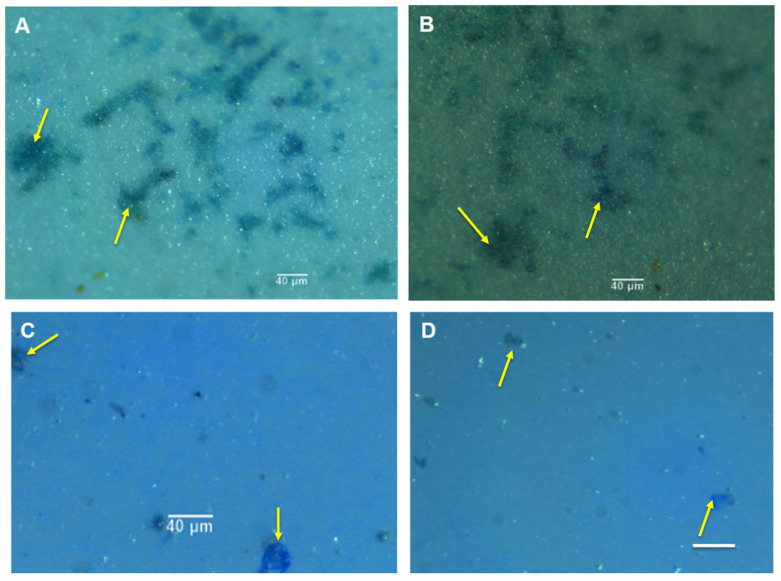
MGP from off the Northumberland coast (North Sea), visualised using bright-field microscopy with fibre light. The MGPs were stained with Alcian blue dye ((**A**,**B**); yellow arrows) and with Coomassie blue ((**C**,**D**); yellow arrows). Scale bar = 40 µm. Images captured using upright Leica microscope and fibre light with 10× objective.

**Figure 3:**
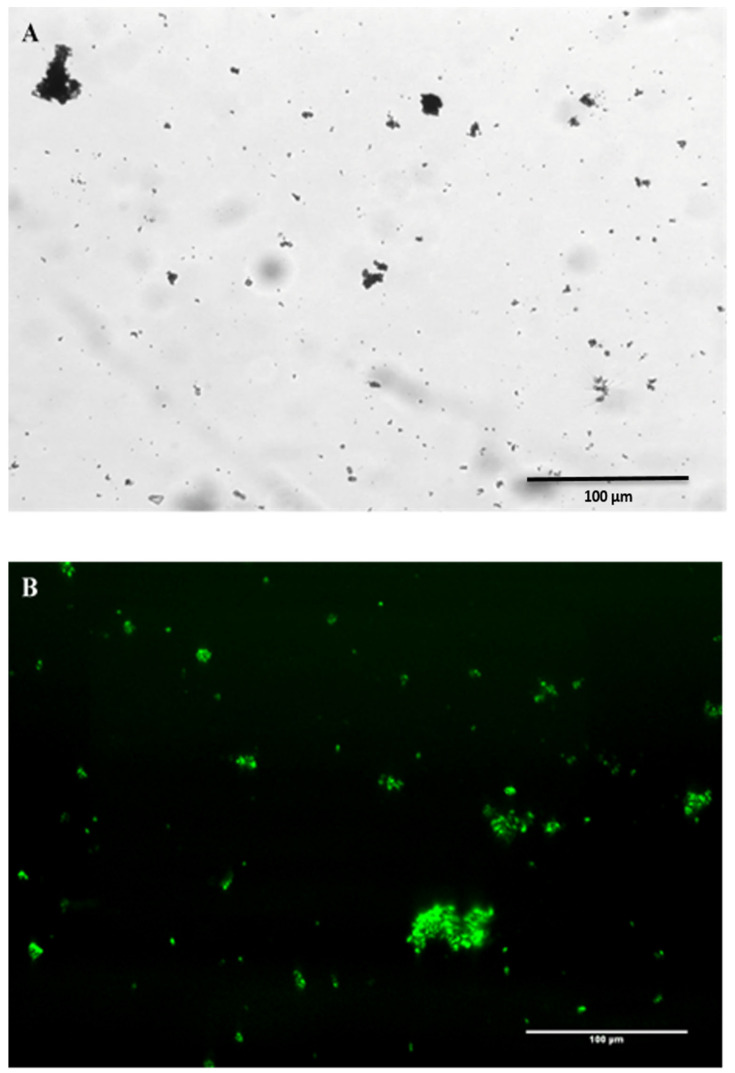
Natural MGPs of different sizes from the North Sea. Bright-field microscopy images of MGPs (**A**) Nucleic acid SYTO 9 green stain of MGP revealing bacterial presence. Fluorescent staining with DNA dye SYTO 9 (1 nM) (**B**). Scale bar = 100 µm. Image capture using 20× objective.

Alcian blue effectively stained EPS in the MGP matrix on PC filters (Figure 2A,B). The MGPs appeared to bind to both Alcian blue and Coomassie brilliant blue (Figure 2C,D). The MGPs visualised using bright-field microscopy (Figure 3A) have sizes from 0.8 µm to 200 µm. Staining MGPs with SYTO 9 (green), a live/dead bacterial DNA stain, revealed the presence of microorganisms (Figure 3B). As seen from the green fluorescence in the images, large aggregates are intensively colonised by microorganisms, with some appearing as single bacterial cells suspended in the sample.

### 2.3. Visualisation of eDNA in Natural MGP Matrix

**Figure 4:**
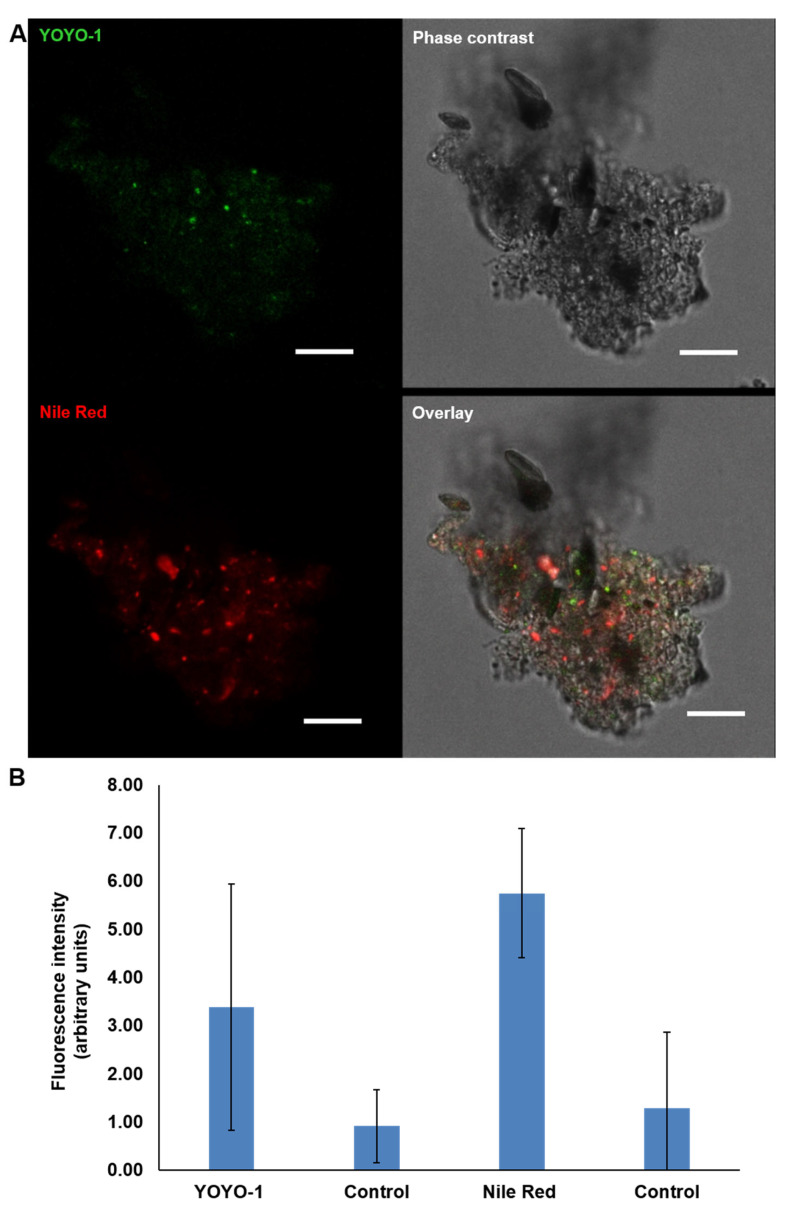
CLSM images acquired using Leica SP8 of natural MGPs stained with YOYO-1 to localise the presence of eDNA (green). Nile red (red) was used as a counter stain for bacterial cell membranes. Scale bar = 5 µm (**A**). Image captured using 63× objective. Quantification of the fluorescence intensity of each fluorophore against the control (unstained MGPs) (**B**). The graph presents the mean ± SD of 15 CLSM micrographs. Error bars represent the standard deviation of the data set (*n* = 3), *p* < 0.05.

CLSM images demonstrated the localisation and presence of eDNA in the matrices of natural MGPs collected from the North Sea. This was confirmed via two independent sets of fluorescence staining that were applied separately to investigate the occurrence of eDNA in MGPs. Natural MGP stained with YOYO-1 and Nile red revealed eDNA (green) and cell membrane (red) and the distribution of the stains was clear in the overlay image (Figure 4A,B).

**Figure 5:**
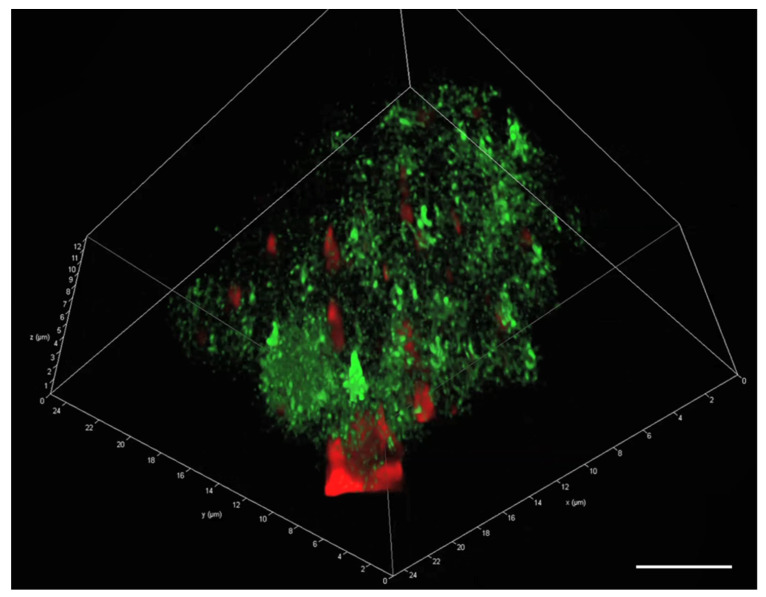
A 3D image of a single natural MGP stained with YOYO-1 (green = extracellular DNA) and Nile red (red = cell membrane). Scale bar = 5 µm. Image captured with Leica SP8 CLSM.

Three-dimensional imaging of a solo natural MGP showed a wide distribution of eDNA (green) throughout the whole particle (Figure 5). The YOYO-1 stain (green) appeared at different levels of brightness, showing the spread of eDNA throughout the particle. On the other hand, the cell membrane stained with Nile red (red) bound only to limited regions of the particle, indicating that the cell membrane occupied larger patches.

**Figure 6:**
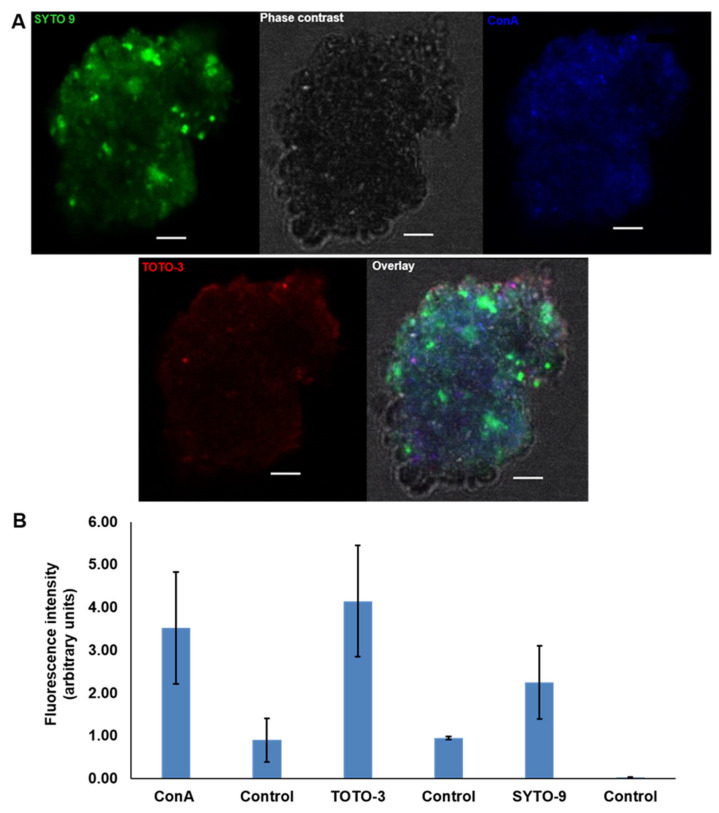
CLSM images of natural MGPs stained with TOTO-3 (red) to localise the presence of eDNA, SYTO 9 (green) for intracellular DNA and ConA (blue) for glycoproteins. Scale bar = 5 µm (**A**). Images acquired with Leica SP8 using 63× objective. Quantification of the fluorescence intensity of each fluorophore against the control (unstained MGP) revealed high mean value of TOTO-3 indicating the presence of eDNA (**B**). The control is unstained particles. The graphs present the mean ± SD of 15 CLSM micrographs. Error bars represent the standard deviation of the data set (*n* = 3), *p* < 0.05.

The visualisation of the MGP matrix components using CLSM showed that TOTO-3 (eDNA, red) has the highest fluorescence intensity of the triple staining set that also contains Con A (glycoproteins, blue) and SYTO 9 (iDNA, green) (Figure 6A,B).

**Figure 7:**
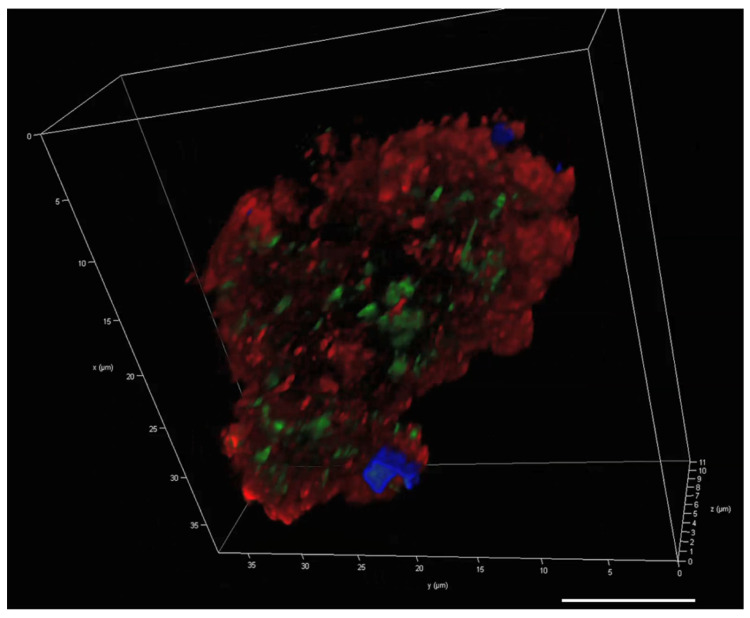
A 3D image of a single natural MGP stained with ConA (blue), TOTO-3 (red)-stained eDNA and SYTO 9 (green)-stained iDNA. Scale bar = 10 µm. Image captured with Leica SP8 CLSM.

The 3D image of a single MGP stained with the same stains (Figure 7), showed a distinct distribution for each component. The coverage of glycoproteins was less than that of DNA and eDNA. The eDNA appears to be widely spread throughout the particle, exceeding the iDNA distribution.

### 2.4. A Pseudoalteromonas atlantica MGP Model

**Figure 8:**
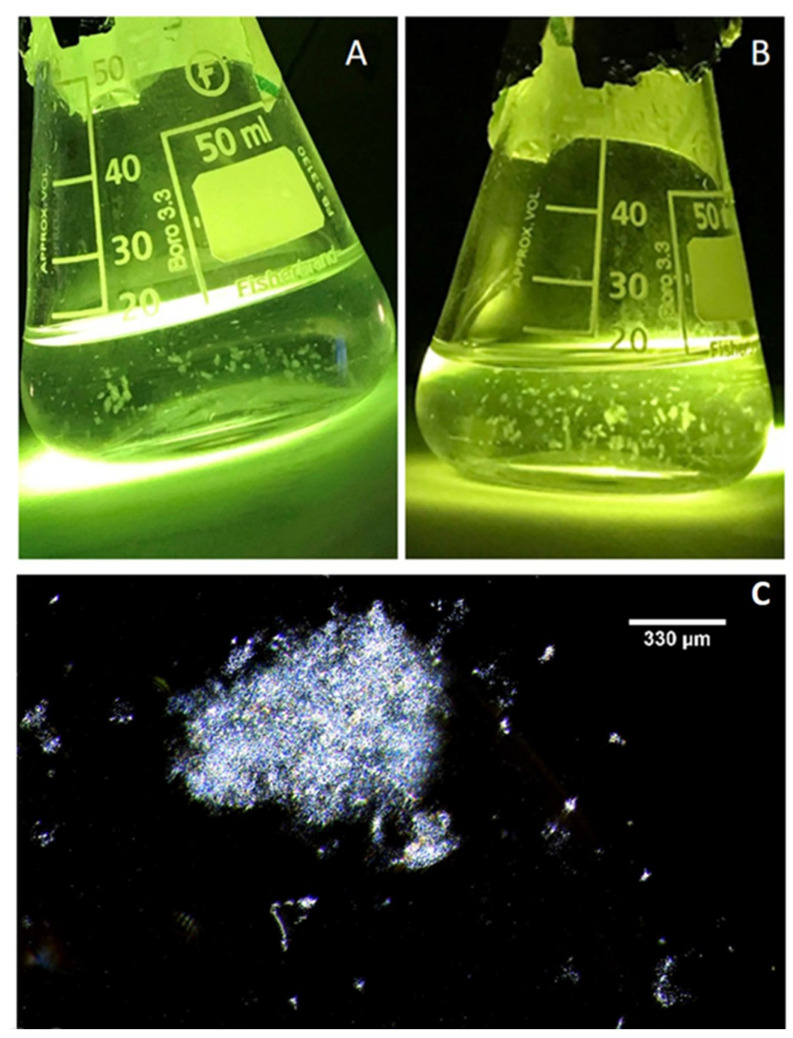
*Pseudoalteromonas atlantica* biopolymers as MGP representative grown in artificial seawater after 96 h (**A**) and after 168 h (**B**). *P. atlantica* particles captured with inverted microscope using 20× objective. Scale bar = 330 µm (**C**).

The EPS particles were clearly observed after 96 h of incubation (Figure 8A), and they evidently aggregated and grew into larger clumps over time (Figure 8B). The *P. atlantica* (PA MGPs) were imaged using dark-field microscopy (Figure 8C), and consisted of relatively clear matrix particles.

### 2.5. Visualisation of the P. atlantica MGP Model Matrix

**Figure 9:**
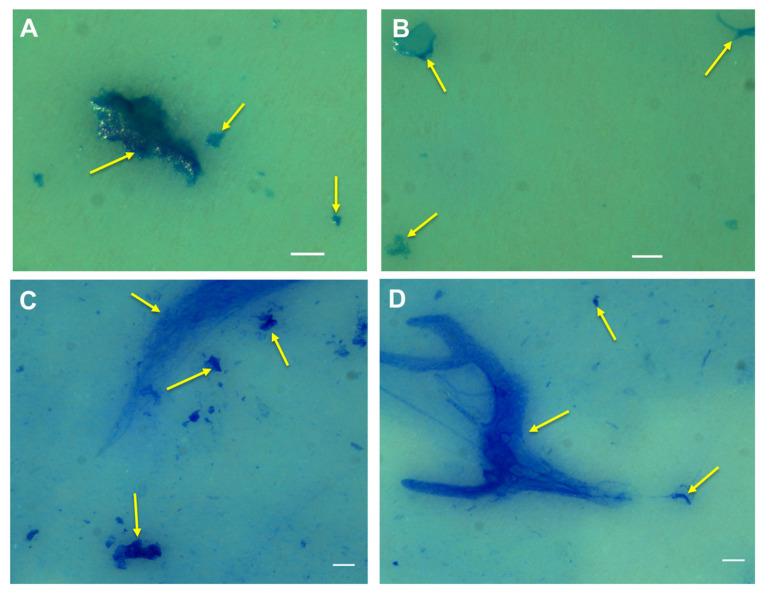
*P. atlantica* EPS stained with Alcian blue ((**A**,**B**); yellow arrows) and Coomassie blue ((**C**,**D**); yellow arrows). Images taken using an upright Leica microscope. Scale bar = 50 µm.

Staining the PA MGPs on PC filters with Alcian blue showed that particles of different sizes were successfully labelled with this dye (Figure 9A,B). Coomassie brilliant blue staining showed visible particles, as presented in Figure 9C,D. However, the Alcian-blue-stained particles here have discrete shapes when compared to CSP-stained particles.

### 2.6. Visualisation of eDNA in P. atlantica EPS (MGP Model)

**Figure 10:**
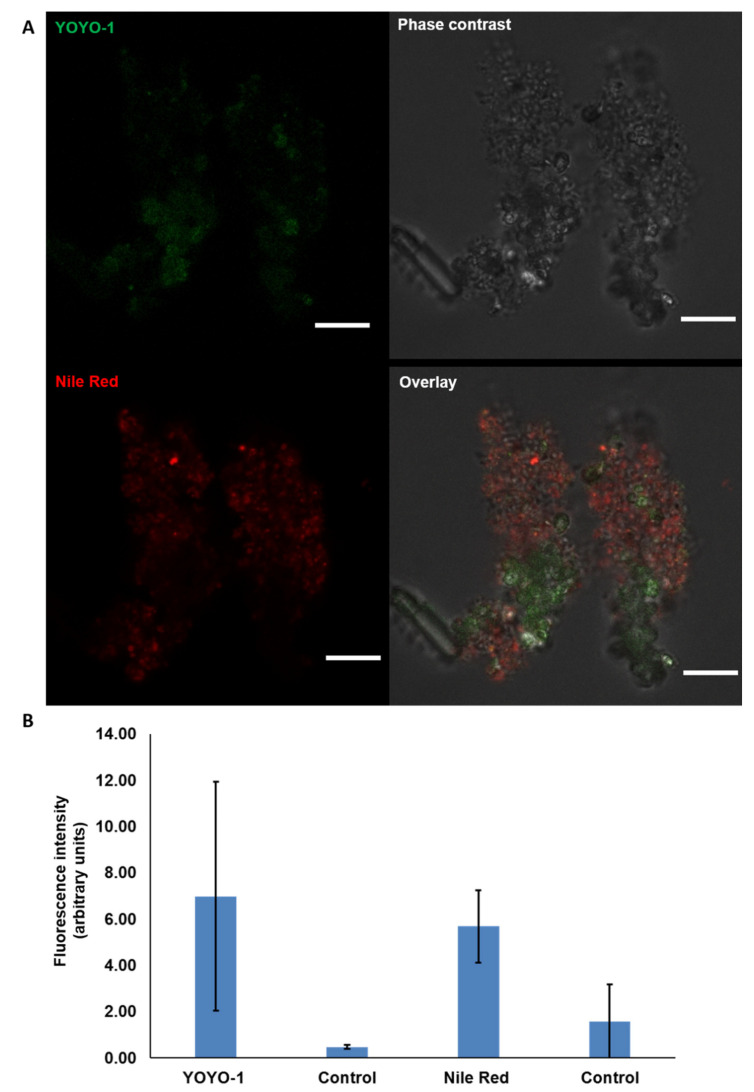
CLSM images acquired using Leica SP8 at 63× of *P. atlantica* EPS MGP model stained for eDNA with YOYO-1 (green); Nile red (red) as counter stain for bacterial cell walls to localise the presence of eDNA. Scale bar = 5 µm (**A**). Quantification of the fluorescence intensity of each fluorophore revealed high mean value of YOYO-1, indicating the presence of eDNA. The control is unstained particles. The graphs present the mean ± SD of 15 CLSM micrographs. Error bars represent the standard deviation of the data set (*n* = 3), *p* < 0.05 (**B**).

**Figure 11:**
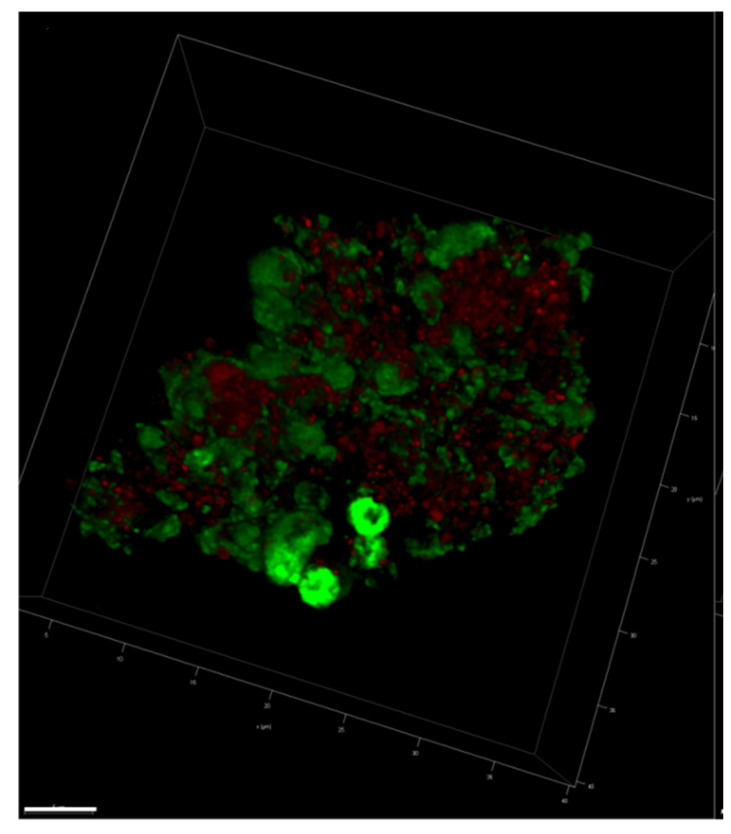
A 3D image of *P. atlantica* particles stained with YOYO-1 (green) for eDNA and Nile red (red). Scale bar = 5 µm.

Using CLSM, the detection of eDNA was probed by cell-impermeant fluorescent dye YOYO-1 combined with Nile red for the cell membrane. The staining showed a clear distribution of green fluorescence indicative of eDNA and red fluorescence for cell membrane proteins in the MGP model. Clear variation can be seen in the brightness of the green and red fluorescence (Figure 10A). The majority of the YOYO-1-stained material was clearly external to the cells and spread throughout the matrix. There was minimal overlap with Nile red, as shown in the overlay image. Compared to YOYO-1, Nile red covered different areas of the aggregate matrix and stained slightly different features. A 3D image of PA MGPs (Figure 11) shows that the distribution of the stains seems to be spreading into more regions in the single particle PA MGP model.

**Figure 12:**
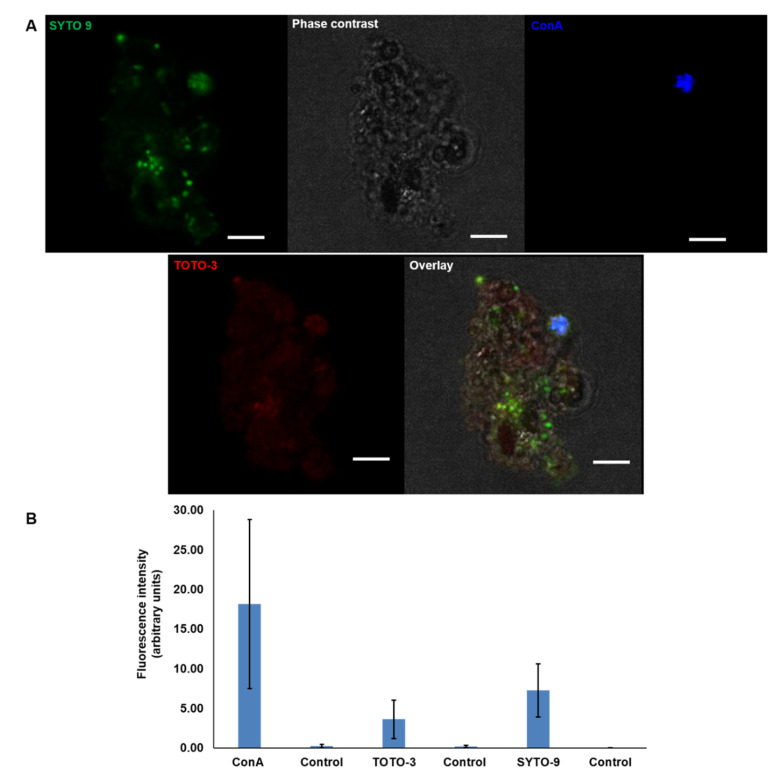
CLSM images acquired using Leica SP8 of *P. atlantica* EPS as MGP model stained for eDNA with TOTO-3 (red) and SYTO9 as counterstain (green), and ConA for glycoproteins (blue). Scale bar = 5 μm (**A**). Quantification of the fluorescence intensity of each fluorophore revealed a high mean value. The control is unstained particles. The graphs present the mean ± SD of 15 CLSM micrographs. Error bars represent the standard deviation of the data set (*n* = 3), *p* < 0.05 (**B**).

**Figure 13:**
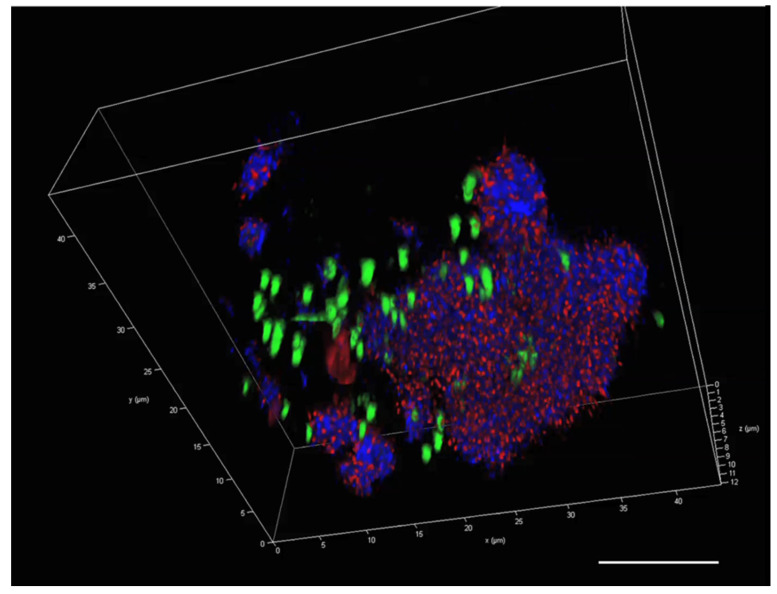
A 3D image of a single particle of *P. atlantica* particles stained with ConA (blue), TOTO-3 (red) and SYTO 9 (green). Scale bar = 10 µm.

The visualisation and quantification of PA MGP matrix components using CLSM is shown in Figure 12A,B, showing that Con A had the highest fluorescent intensity of the triple staining set. Visualisation of the 3D single PA MGP particles (Figure 13) showed a clear distribution of the components with the dominance of Con A (glycoproteins, blue). TOTO-3 (eDNA, red) appeared scattered in association to the matrix, whereas SYTO 9 (green) appeared in larger spots that may represent regions of recent cell lysis.

## 3. Discussion

This study focused on MGPs in the size range 0.4–100 µm that are thought to constitute the “glue” which binds sinking marine aggregates together [8,23,50]. In addition, we aimed to better understand the presence of eDNA in smaller (and possibly younger) MGPs prior to any further growth and/or the increased structural complexity typical of larger flocs, and prior to them becoming a surface-attached biofilm. Therefore, a laboratory model system for studying MGPs made up of *P. atlantica* flocs was employed to explore their composition, in parallel to studying naturally occurring and biochemically much more complex MGPs isolated from seawater. The in vitro staining of both particle types with specific eDNA stains visualised via CLSM revealed the presence of eDNA within MGPs for the first time. With these two staining approaches, it was possible to differentiate between intracellular and extracellular DNA in the MGP matrices. Despite the possibility that impermeant stains might penetrate damaged microorganism cells attached to the MGPs, the staining is generally effective for labelling eDNA [51].

### 3.1. Isolation of MGPs in Liquid Suspension

Developing a method to study MGPs in liquid suspension rather than affixed to a filter membrane [20,31,32] was a pre-requisite to enable their study and characterisation in a form resembling their natural occurrence in the oceans. A particular strength of the method presented here lies in the technique of isolation that maintains the three-dimensional structure of the MGPs and permits their study in 3D form. A similar method was used to investigate marine snow particles (in situ), although they were larger than 500 µm in diameter [42]. Although fragile, MGPs adhere well to various surfaces and can also self-aggregate in solution over time. While this may hinder the recovery of the particles from the filters, we found that the particles could be recovered from PC filters quite easily. As PC filters are smooth and hydrophobic, they facilitate capturing suspended particles with negligible adsorption to the filters, enabling the full retrieval of particles and their re-suspension in artificial seawater [32,52,53]. Although numerous different methods and tools have been used to sample and collect MGPs, few studies have examined MGPs in their natural form (liquid suspension) [31,52,53].

### 3.2. The Presence of eDNA in Natural MGPs and P. atlantica MGPs

The conventional staining methods for the study of MGPs [20,24,42,54] show that both natural MGPs and those derived from *P. atlantica* can be classified as diverse mixtures of particles that include TEP and/or CSP [23]. Investigations of the matrix composition of MGPs is challenging due to their heterogeneity and structural complexity. Their heterogeneity is attributed to their having a range of various sources including bacterial EPS, microalgae such as diatoms, and macroalgae [11,55]. Previously the stains used here were reported to label eDNA in biofilms and bacterial aggregates [19,52,56,57], and CLSM imaging has been used in the exploration of marine and freshwater aggregates and activated sludge [58,59,60,61]. By combining fluorescent dyes with bioimaging in our work, we were able to validate the occurrence of eDNA associated with MGP. Marine snow has previously been investigated using fluorescent dyes and CLSM [42,62]. Holloway and Cowen [41] characterised the compositional structure of marine snow in terms of investigating the presence of polysaccharides using Concanavalin A, proteins using [5-(4,6-dichlorotriazin-2-yl] amino) fluorescein, and intracellular DNA using propidium iodide. However, they did not explore eDNA in marine snow structures.

The bacterial *P. atlantica* MGP model used in this study generally revealed similar labelling patterns to natural MGPs. The fluorescent staining protocols successfully stained and localised the eDNA in the MGP matrix and the *P. atlantica* MGPs. The variation in the intensity of the fluorophore may be due to the different concentrations and residence times of eDNA in the MGP matrices. These variations may also be attributable to the age of the MGPs (something that is not yet easy to determine), and other external factors such as temperature, pH, the presence of bacterial consumers of DNA, and dissolved enzymes [63,64,65]. As a natural gel, the possibility of autofluorescence in the control samples from various natural pigments such as chlorophylls and carotenoids cannot be discounted. In addition, the association of eDNA with proteins (e.g., packaging proteins) and histones could hinder DNA degradation and preserve the DNA for longer within the milieu [66,67].

Assuming eDNA may accumulate through time in the MGP matrices, the constant release of eDNA from living bacterial cells residing in the MGP is possible. An implication is that eDNA availability may be linked to bacterial community abundance. Indeed, some bacteria may consume DNA as a source for phosphorus and nitrogen [68,69]. However, eDNA could also attach to MGPs from the surrounding environmental DNA of the seawater, by several mechanisms [70,71,72]. Trials in which DNase I and MNase (Micrococcal DNase) were added to MGP and *P. atlantica* particle suspensions, as investigated with 18-24 h time-lapse imaging, resulted in larger particle dispersion to smaller fragments with more motility observed under the microscope after 24 h (Supplementary Data). Furthermore, a study on the DNase digestion of marine DNA showed that around 3–8% of the marine DNA is DNase resistant and cannot be digested by microorganisms [73]. Thus, eDNA within MGPs may be bound to proteins that shield it from DNase degradation [74,75,76]. This prompts a need a for further studies to examine different DNase effects on MGPs at different stages of aggregation. Furthermore, evidence of the co-occurrence of extracellular deoxyribonuclease (DNase) activity of marine bacteria within MGPs has also been presented [77]. Although it is possible to sequence eDNA, one significant problem is the difficulty in separating naturally occurring eDNA from the DNA inside bacterial cells. Little is currently known about the DNA sequences of eDNA from MGPs; studies on eDNA in biofilms from other studies have generally shown that there is little difference between the sequences of DNA and bacterial chromosomal DNA. The sequencing of marine particles has however been reported, for example, when tracking the species composition of sinking particles [78,79], although the identification of bacterial species within natural MGPs was not a focus of this work.

Bioimaging was also employed for the study of marine gels for the first time using a combination of fluorescent stains (with no overlap) that simultaneously stained polysaccharides, glycoproteins and eDNA in natural MGPs and *P. atlantica*’s MGPs models. These were not previously applied to probe eDNA either in marine particles or in marine snow. However, some studies have investigated marine particles in situ by implementing video and holographic techniques [42,80,81]. The characterisation of marine snow (>500 µm) composition in situ implemented soft embedding and the sectioning of microbial diversity using fluorescent in situ hybridisation (FISH)-probe labelling. This involved multiple stains, including Alcian blue, Coomassie brilliant blue and Concavalian A at high resolution epifluorescence and CLSM, although eDNA was not specifically targeted [42].

A recent study characterised three different groups of dissolved DNA (D-DNA); vesicles, viruses and exocellular Free-DNA at different depths of the North Pacific Subtropical Gyre. This revealed that microbial D-DNA in the ocean originates in the euphotic zone via viral lysis and is subsequently transported downward into the mesopelagic zone associated with organic matter [73]. A clear link between MGP and organic matter transfer to the deep ocean is thus established. Even so, the extent to which eDNA is implicated in altering the structural integrity of MGPs and in their aggregating of MGPs remains to be addressed. This is a key research question in the marine gels and bioflocs domain that has implications for marine snow formation and dynamics, including its conversion from labile to recalcitrant forms that subsequently are involved in long-term carbon sequestration via sedimentation. In addition, eDNA has been shown to inhibit the growth of the marine microalga *Nannochloropsis gaditana* [82], further illustrating the complex nature of its interactions in microbial ecology. Clearly, additional research is required to further understand the impact of eDNA on different sizes, ages and labilities of carbon within MGPs. Another aspect is that the microbial communities within MGPs may use eDNA as a source of carbon and nitrogen using nucleases. This may also influence the stability of MGPs [77,83].

## 4. Conclusions

This study set out to develop a new method for isolating MGPs as suspended particles, rather than the more traditional methods which use immobilization onto filter paper, and to investigate their composition in terms of the presence or absence of eDNA. In addition, a bacterial EPS model of *P. atlantica* was used to mimic a natural MGP to understand the initial composition of eDNA in newly produced bacterial MGPs. There is strong evidence for a significant contribution from eDNA to MGP. This is an important finding that highlights important roles for MGPs in the formation and composition of marine snow, sinking aggregates, and sediments. Consequently, MGPs play a potentially important role in the marine carbon budget. Improving our understanding of eDNA in MGPs will rely on a better appreciation of the molecules with which eDNA forms complexes. A combination of advanced imaging and quantification techniques, used in conjunction with appropriate MGP models, will be necessary to achieve such objectives. Future work should aim to more completely elucidate the importance of eDNA in the structural integrity of MGPs and in the dynamics of labile and recalcitrant forms of particulate organic matter. Our findings confirm the biochemical heterogeneity of MGP composition, and question the ability of two commonly used stains to differentiate between two types of gel particles. Additionally, understanding the precise biochemical compositions of MGPs will be important for elucidating their contributions to marine ecology and global biogeochemistry.

## 5. Materials and Methods

### 5.1. Seawater Collection

North Sea surface seawater samples were collected at 10–20 cm depth in sterilised Nalgene bottles (5 and 10 L) deployed overside from RV *Princess Royal*. Sampling was approximately one mile offshore from the NE UK coast (55°06.972 N, 1°25.600 W) on 23 March 2015, 23 February 2016, 18 July 2016, 31 October 2016, 13 February 2017 and 25 March 2018. Samples were transferred to cold laboratory storage (4 °C cold room) within three hours of collection.

### 5.2. Isolation of Natural MGPs from Seawater by Filtration

MGPs typically range from 0.4 to 100 µm in diameter [84]. Samples were therefore prefiltered through a 100 µm stainless steel mesh to remove larger particles and organisms. Subsequent filtration through 0.4 µm hydrophobic polycarbonate (PC) membranes, (Whatman, GE, USA) was carried out using a glass vacuum filtration system (47/50 mm, https://www.sartorius.co.uk/, accessed on 20 February 2016). The vacuum was set to a maximum of 150 mm Hg to ensure the collection of intact MGPs [31]. To avoid filter clogging, each PC filter was replaced with a new one for every 1 L of seawater filtered.

### 5.3. Isolation of Concentrated Natural MGPs in Suspension

Following filtration, the PC filters were placed into sterile 50 mL polypropylene falcon tubes containing 5 mL of 0.2 µm filter sterilised artificial seawater (Millipore Syringe Filters, Cole-Parmer, Darmstadt, Germany). The MGPs were released from the PC filters with gentle shaking and rolling and the filters were then removed to leave suspensions of MGPs in sterile seawater. These were refrigerated until further use.

### 5.4. Bacterial Strains and Growth Conditions

Single species of bacteria were explored for their suitability as model systems for studying artificial MGPs. Three marine bacterial strains, *Pseudoalteromonas atlantica* (DSM-No. 6840), *Pseudoalteromonas citrea* (DSM-No. 8771) and *Idiomarina homiensis* (DSM-No. 17923) were selected based on their reported ability to produce gel polymers [47,48,49]. All were obtained from the German Collection of Microorganisms and Cell Cultures (DSMZ: https://www.dsmz.de/). Following cultivation in standard marine broth (Difco 2216, Becton Dickinson, UK), each was grown on marine agar plates (Difco marine agar 2216, Becton Dickinson, UK), with incubation at 20 °C for *P. atlantica*, 26 °C for *P. citrea* and 28 °C for *I. homiensis*. *P. atlantica* showed the highest polymer production (based on CFU) and was therefore selected as the most suitable for further use.

### 5.5. A Model MGP Using Pseudoalteromonas atlantica

In sterile 50 mL Erlenmeyer flasks, a single colony of *P. atlantica* was inoculated into 20 mL of 0.2 µm filtered artificial seawater (ASW; 33 g/L (Sea salts: Sigma-Aldrich, Gillingham, UK); final salinity 33–34) and incubated at room temperature with agitation at 150 rpm using a temperature-controlled orbital shaker (Stuart^TM^, Nottingham, UK).

### 5.6. Compositional Analysis of Natural MGPs and a P. atlantica MGP Model

The composition of both natural and *P. atlantica* model MGPs was evaluated using multiple staining techniques. Staining for acidic polysaccharides followed Alldredge, Passow [20], with some modification of washing the filters using sterilised artificial seawater. Particles were stained on 0.4 µm PC filters with 0.2 µm-filtered 0.02% Alcian blue (Sigma-Aldrich) dissolved in 0.06% (*v*/*v*) acetic acid (pH 2.5) for 1 min, washed with ASW three times to eliminate excess dye, and gently vacuum filtered as described previously. Coomassie brilliant blue G (CBBG, Sigma-Aldrich), was used [85], with modification of the washing step: particles were washed with ASW three times to eliminate excess dye, followed by gentle vacuum filtration as above.

### 5.7. MGP Staining with eDNA Probes

Two staining techniques were used to probe for eDNA in both natural MGPs and *P. atlantica* model MGPs. Initially, a combination of fluorescent stains, YOYO-1 (Life Technologies, Burlington, ON, Canada) and Nile red (Sigma-Aldrich, St. Louis, MO, USA), was used. YOYO-1 is an ultrasensitive impermeant dye with a high affinity for nucleic acid that is commonly used to target eDNA [86]. Nile red is a lipophilic stain for intracellular lipid used widely as a membrane dye [87]. Here, Nile red was applied as a counter stain to differentiate between the bacterial cell membranes and eDNA found in the MGPs.

In 2 mL sterile Eppendorf micro-centrifuge tubes, 500 µL of the suspended particles were stained with YOYO-1 [86] at a concentration of 2.4 nM for 10 min at 20 °C and covered in foil to exclude light. Nile red was then added at a concentration of 1 µg/mL for 15 min, covered with foil and dark-stored prior to microscopy.

Another set of tri-stains was used, combining Concanavalin A (Con A, Tetramethylrhodamine Conjugate A, C860, Life Technologies), a lectin stain used here to characterise glycoproteins and carbohydrates [88], TOTO-3 (T3604, Life Technologies), a cell-impermeant DNA stain used for eDNA probing, and SYTO-9 (Life Technologies), a cell permeant DNA stain for nucleic acid of live and dead bacterial cells. TOTO-3 and SYTO 9 are commonly applied together to distinguish between intracellular and extracellular DNA [19].

In 2 mL sterile tubes, 500 µL of the suspended particles were stained with 10 µM Con A (stock concentrations 1 mM), 2 µM TOTO-3 (stock concentrations 1 mM) and 20 µM SYTO 9 (stock concentrations 5 mM). Stains were added to 500 µL of the natural MGPs or *P. atlantica* MGPs in the same manner. The labelled samples were incubated in the dark for 15 min at room temperature prior to bioimaging to bind to substrate and to avoid photo destruction [89].

### 5.8. MGP Visualisation by Microscopy

Samples were placed in a sterile µ-Slide with 8 wells and a glass bottom (ibidi Treat 180215/1; Ibidi, https://ibidi.com/, accessed on 15 March 2016). A volume of 250 µL of each sample was placed in each well. This volume was chosen to minimise the sample thickness inside the wells and to unify the focal plane. A Leica TCS SP2 confocal microscope (Leica Microsystems CMS GmbH, Germany, www.leica-microsystems.com) was used for the microscopic examination of MGPs. Images were captured at different objective sizes.

### 5.9. Bioimaging

A Leica SP8 high-resolution fluorescent inverted microscope with water immersion (www.leica-microsystems.com) was used for the CLSM of the stained MGPs. The lens used was model HCPL APO CS2, with an objective 63×/1.20 numerical aperture (NA) [90]. The fluorophore was stimulated using a white light laser (WLL), where each fluorophore was excited and emitted (acquired) at certain suitable wavelengths (Table 1). The aggregate matrix staining was evaluated based on the binding and visibility of the dyes.

### 5.10. Quantification of Fluorescent Dye Intensity 

The fluorophores in the CLSM-acquired images were quantified using LAS X software (Leica microsystems), where the mean values of the fluorescent intensity were measured by quantifying the area of the particle. The mean intensity values of each channel (fluorophore acquisition) were compared to the control, i.e., the unstained particles. The averages of five particle replicates were measured and the experiments were repeated three times. The average mean values of the fluorescence intensity of 15 randomly selected particles were quantified per stain, repeated in three experiments (mean ± SD; *p* < 0.05), and compared between stained and unstained particles using an unpaired t-test.

## Figures and Tables

**Table 1:** List of fluorophores and the excitation and acquisition wavelengths of visualisation.

Fluorophore	Target	Excitation (nm)	Acquisition (nm)
YOYO-1	eDNA	491	501–532
Nile Red	Cell membrane	553	567–781
Concavalian A (TMR)	Glycoproteins	552	560–643
TOTO-3	eDNA	642	652–784
SYTO-9	DNA	483	490–591

## Data Availability

Not applicable.

## Supplementary Materials

The following supporting information can be downloaded at: https://www.mdpi.com/article/10.3390/gels9030251/s1, Figure S1: The first observations of the effect of DNase I on natural MGP 0-24 h (A) and after 120 h (B); Figure S2: MATLAB analysis of marine aggregates incubated in sterile MilliQ water as a control (A) and in DNase I (B); Figure S3: Filtered seawater at 0.4 µm incubated with bacterial DNA 1 mg/mL; Figure S4: *P. atlantica* EPS (MGP model) in artificial seawater; Figure S5: *P. atlantica* EPS (MGP model).
