# Supplementary material for: Isolation and Staining Reveal the Presence of Extracellular DNA in Marine Gel Particles
Source: Gels. 2023 Mar 21;9(3):251. doi: 10.3390/gels9030251 (PMC10048003; doi:10.3390/gels9030251)
Supplement: Supplementary file 1 [file gels-09-00251-s001.zip › gels-2268280-supplementary.pdf]

Supplementary Figures

I. The effect of nucleases on the disintegration of natural MGPs

DNase I against Natural MGPs

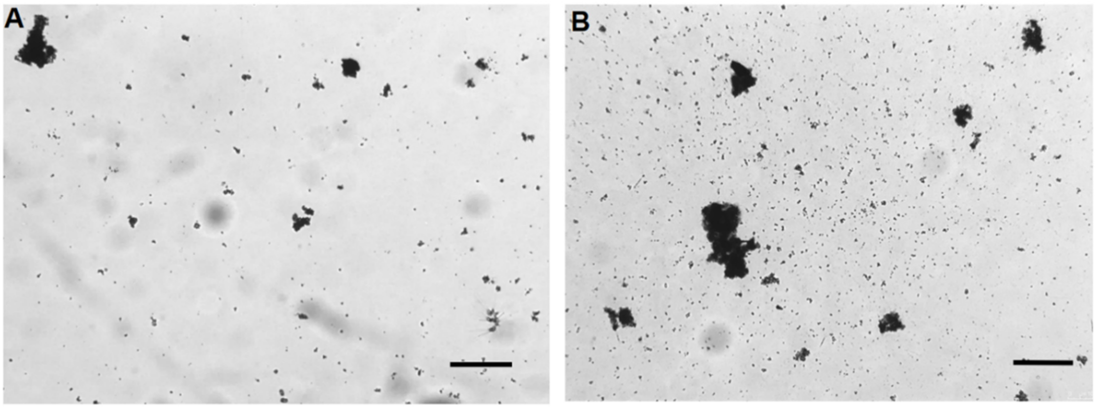

Supplementary Figure S1. The first observations of the effect of DNase I on natural MGP 0-24 hours (A) and after 120 hours (B). Scale bar represents 50 μm.

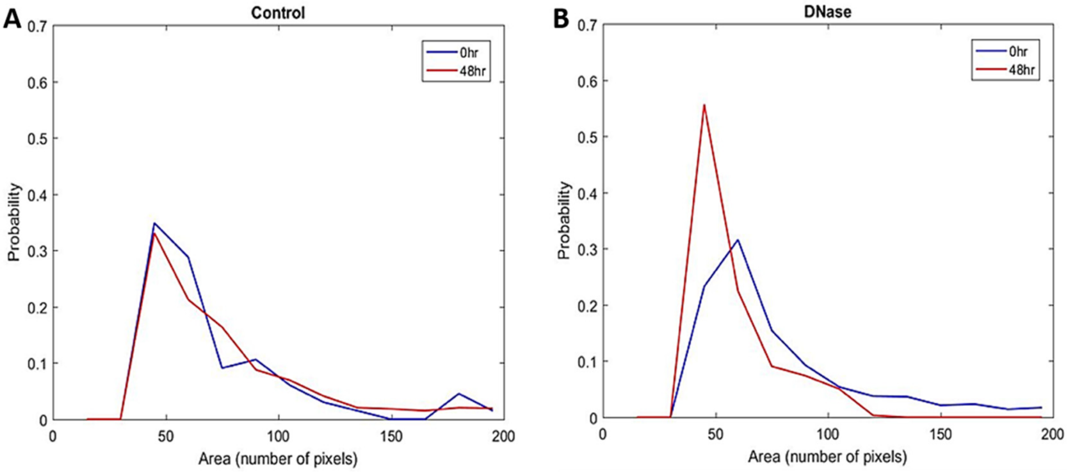

Supplementary Figure S2. MATLAB analysis of marine aggregates incubated in sterile MilliQ water as a control (A) and in DNase I (B).

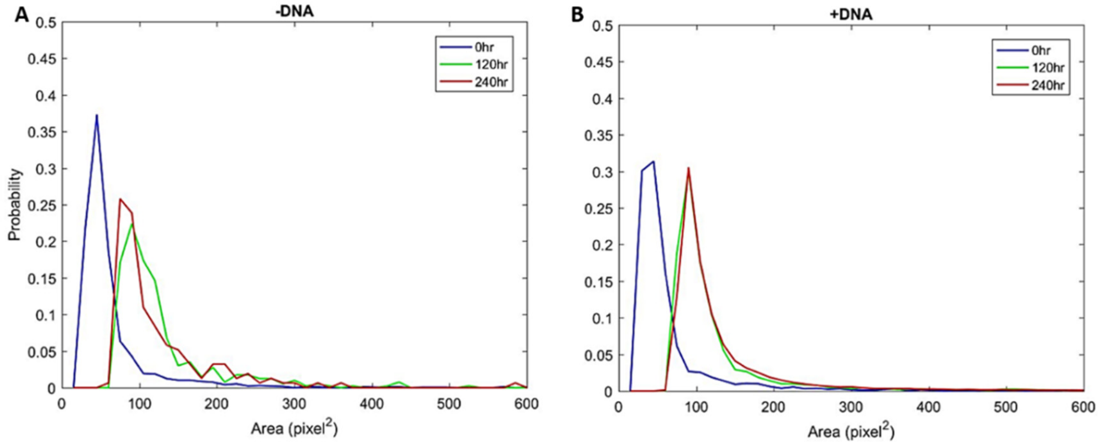

Supplementary Figure S3 Filtered seawater at 0.4 µm incubated with bacterial DNA 1 mg/ ml.

1. MNase against natural MGPs

A time-lapse image using CLSM (Leica SP8) was acquired to investigate the dispersal of MGP by the addition of MNase (2x 10^6 gel U) at 37°C. This resulted in no change in the particles over the 18-hour experiment. This would be due to deactivation of the enzyme by seawater salts.

2. MNase against *Pseudoalteromonas atlantica* EPS

Preliminary results of the effect of MNase on the dispersal of *P. atlantica* EPS aggregates.

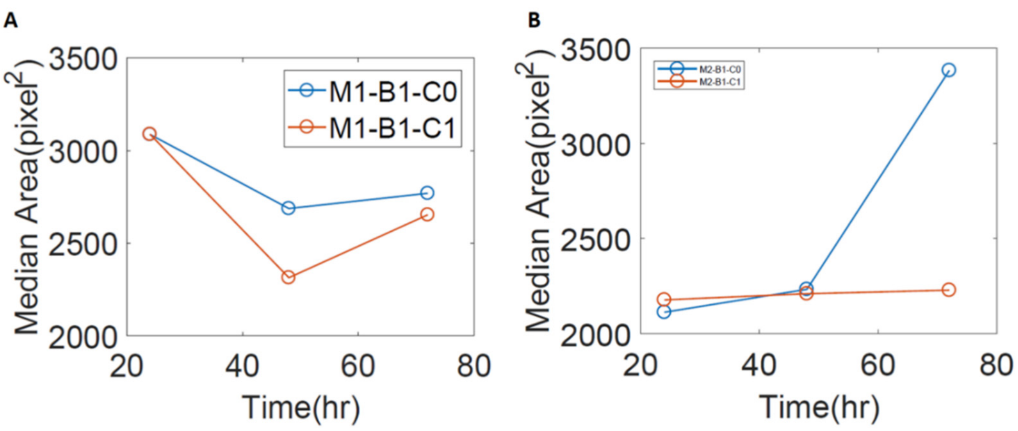

Supplementary Figure S4. *P. atlantica* EPS (MGP model) in artificial seawater incubated with C0= no enzyme and C1= 200 gel U/ ml (A) and *P. atlantica* EPS (MGP model) incubated in 0.2 µm filter sterile MilliQ water with C0= no enzyme and C1= 200 gel U/ ml (B).

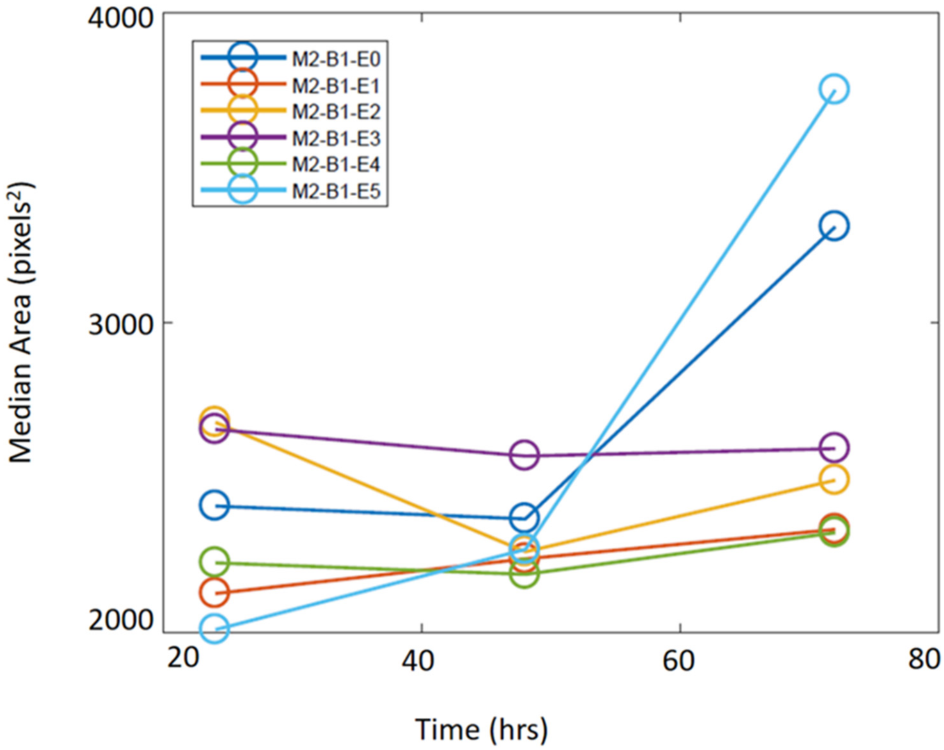

Supplementary Figure S5 *P. atlantica* EPS (MGP model) incubated with: E0= MilliQ water, E1= MNase 2x10^6 gel U/ ml, E2= G22 (supernatant of DNase active bacteria isolated from MGPs), E3= X2 (supernatant of DNase active bacteria isolated from MGPs), E4= AW2 supernatant of DNase active bacteria isolated from marine sediments, E5= *P. atlantica* in 0.2 µm filter sterile MilliQ water.
